# Imaging features of localized *IDH* wild-type histologically diffuse astrocytomas: a single-institution case series

**DOI:** 10.1038/s41598-022-25928-2

**Published:** 2023-01-16

**Authors:** Yuji Kibe, Kazuya Motomura, Fumiharu Ohka, Kosuke Aoki, Hiroyuki Shimizu, Junya Yamaguchi, Tomohide Nishikawa, Ryuta Saito

**Affiliations:** Department of Neurosurgery, Nagoya University School of Medicine, 65 Tsurumai-Cho, Showa-Ku, Nagoya, 466-8550 Japan

**Keywords:** Neurology, Neurological disorders, Oncology, Surgical oncology

## Abstract

Isocitrate dehydrogenase wild-type (*IDH*wt) diffuse astrocytomas feature highly infiltrative patterns, such as a gliomatosis cerebri growth pattern with widespread involvement. Among these tumors, localized *IDH*wt histologically diffuse astrocytomas are rarer than the infiltrative type. The aim of this study was to assess and describe the clinical, radiographic, histopathological, and molecular characteristics of this rare type of *IDH*wt histologically diffuse astrocytomas and thereby provide more information on how its features affect clinical prognoses and outcomes. We retrospectively analyzed the records of five patients with localized *IDH*wt histologically diffuse astrocytomas between July 2017 and January 2020. All patients were female, and their mean age at the time of the initial treatment was 55.0 years. All patients had focal disease that did not include gliomatosis cerebri or multifocal disease. All patients received a histopathological diagnosis of diffuse astrocytomas at the time of the initial treatment. For recurrent tumors, second surgeries were performed at a mean of 12.4 months after the initial surgery. A histopathological diagnosis of glioblastoma was made in four patients and one of gliosarcoma in one patient. The initial status of *IDH1*, *IDH2*, *H3F3A*, *HIST1H3B*, *and BRAF* was “wild-type” in all patients. *TERT* promoter mutations (C250T or C228T) were detected in four patients. No tumors harbored a 1p/19q codeletion, *EGFR* amplification, or chromosome 7 gain/10 loss (+ 7/ − 10). We assessed clinical cases of localized *IDH*wt histologically diffuse astrocytomas that resulted in malignant recurrence and a poor clinical prognosis similar to that of glioblastomas. Our case series suggests that even in patients with histologically diffuse astrocytomas and those who present with radiographic imaging findings suggestive of a localized tumor mass, physicians should consider the possibility of *IDH*wt histologically diffuse astrocytomas.

## Introduction

Gliomas are invasive and diffusely infiltrative tumors of the central nervous system^1^. Grading of gliomas traditionally depends on their morphology and immunohistochemical evaluation. Diffuse astrocytomas and oligodendrogliomas, typically characterized by moderately increased cellularity, small nuclear atypia, low mitotic activity, absence of necrosis and microvascular proliferation, have been classified as grade II according to the World Health Organization (WHO) classification until the 2016 revision of the fourth edition^2^. In the 2016 WHO classification, mutations in isocitrate dehydrogenase (*IDH*) 1 and 2 are key genetic events in adult lower grade gliomas (LrGG), which include WHO grade II and grade III gliomas. Most LrGGs harbor *IDH* mutations, whereas most glioblastomas (GBMs) do not^2^.

There is a rare subgroup of LrGGs that do not harbor *IDH* mutations: *IDH* wild-type (*IDH*wt) gliomas. These tumors are associated with a poorer prognosis and poorer response to treatment than *IDH* mutant-type (*IDH*mut) gliomas^3^. Due to the rarity of *IDH*wt grade II gliomas, most earlier studies combined grade II and III gliomas for analysis^4^. However, some research suggests that *IDH*wt grade II and grade III tumors differ significantly in terms of clinical prognoses and biological behavior^5–8^. In particular, *IDH*wt grade III astrocytomas strikingly resemble *IDH*wt GBMs^6,7^. *IDH*wt grade II astrocytomas, on the other hand, show less malignant clinical features^5,8^.

A recent consensus from the cIMPACT-NOW consortium has proposed that grade II and III *IDH*wt astrocytomas harboring epidermal growth factor receptor (*EGFR*) amplifications, and/or combining whole chromosome 7 gain and whole chromosome 10 loss (+ 7/ − 10), and/or harboring telomerase reverse transcriptase (*TERT*) promoter mutations should be considered true GBMs, given their poor survival prospects^9^. These LrGGs with molecular features of GBMs (i.e., features of molecular GBMs) have almost the same radiographic and histological findings as *IDH*mut LrGGs. The recent (2021) WHO classification of central nervous system (CNS) tumors applies molecular criteria that allow for a diagnosis of GBM, CNS grade 4, of *IDH*wt astrocytic gliomas, even in the absence of high-grade histopathologic features, when at least one of the following molecular features is present: concurrent *EGFR* amplification, chromosome 7 gain/10 loss (+ 7/ − 10), or a *TERT* promoter mutation^10^. Regarding the overall survival (OS) of *IDH*wt diffuse astrocytomas, one group has reported that median OS of the patients with *IDH*wt diffuse astrocytomas meeting the definition for molecular GBM was 42 months, which had no significant difference compared with patients with *IDH*wt diffuse astrocytomas not meeting this definition (median OS: 57 months) ^11^. In contrast, another group has revealed that the median OS for *IDH*wt diffuse astrocytomas, grade 4 (based on the 2021 WHO classification of CNS tumors) and *IDH*wt glioblastomas was similar (23.8 months vs 19.2 months)^12^. Therefore, the exact prognosis of *IDH*wt diffuse astrocytomas remains unknown.

Some studies have reported that localized types of molecular GBMs are rarer than the infiltrative type^13–15^. Moreover, both *IDH*wt grade II astrocytomas and *IDH*wt grade II astrocytomas meeting the definition of molecular GBM (according to the 2016 WHO classification of CNS tumors) were reported to possess highly infiltrative patterns, such as gliomatosis cerebri growth patterns with widespread involvement.^11^ Although localized tumors are typical in LrGGs, it is not clear whether this radiographic feature affects the clinical prognosis of *IDH*wt LrGGs.

The authors analyzed five cases of localized *IDH*wt histologically diffuse astrocytomas and found that they all resulted in malignant recurrence and a poor clinical prognosis similar to that of GBMs. Although a number of studies have investigated *IDH*wt LrGGs^11–13,16,17^, very few studies have reported detailed information on localized *IDH*wt LrGGs. In the present study, we therefore describe the clinical, radiographic, histopathological, and molecular characteristics of localized *IDH*wt histologically diffuse astrocytomas.

## Results

**Figure 1:**
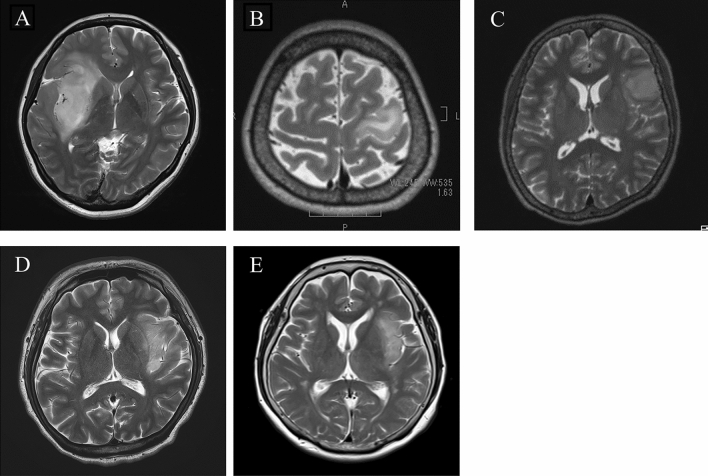
Preoperative axial T2-weighted MRI, showing a high-intensity area with a well-defined tumor border of the right insular tumor (Case 1) (**A**), of the precentral gyrus (Case 2) (**B**), of the left inferior frontal gyrus tumor (Case 3) (**C**), of the left insular tumor (Case 4) (**D**) and, of the left insular tumor (Case 5) (**E**).

**Figure 2:**
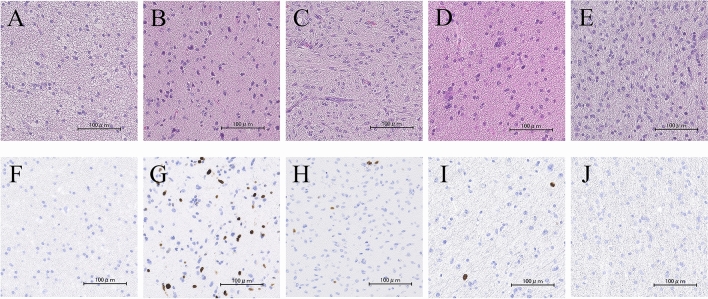
Hematoxylin and eosin staining of the initial surgical specimen showing a diffuse astrocytoma. Case 1 (**A**), Case 2 (**B**), Case 3 (**C**), Case 4 (**D**), and Case 5 (**E**). The Ki-67 labelling index was approximately 2% in Case 1 (**F**), 3% in Case 2 (**G**), 2% in Case 3 (**H**), 5% in Case 4 (**I**), and 2% in Case 5 (**J**). Scale bars: 100 μm.

The five patients’ clinical characteristics are summarized in Table 1. All patients were female, and their mean age at the time of the initial treatment was 55.0 years. The tumors were located in the insula in three patients (60.0%), in the precentral gyrus in one patient (20.0%), and in the frontal operculum in one patient (20.0%). The tumors were located in the left hemisphere in four cases (80.0%) and in the right hemisphere in one case (20.0%) (Fig. 1). All patients had focal disease, not including gliomatosis cerebri or multifocal disease. All initial tumors showed no contrast enhancement on Magnetic Resonance Imaging (MRI). Gross total resection (GTR) was achieved in three patients and partial resection (PR) in two patients. The pathological diagnosis in all cases was diffuse astrocytoma (Fig. 2A–E) with a Ki-67 labeling index of 2–5% (Fig. 2F–J). Thus, no adjuvant chemoradiotherapy was performed.

**Table 1:** Clinical characteristics of five patients with localized *IDH* wild-type histologically diffuse astrocytomas.

Patient No	Age (years)	Sex	Tumor location	hemisphere	Contrast enhancement on MRI	At initial diagnosis			At recurrence			PFS	Status
						Histology	EOR	Adjuvant therapy	Histology	EOR	Adjuvant therapy		
1	39	F	Insula	R	–	DA	GTR	none	GBM	STR	TMZ, RT 60 Gy	7.5	Dead
2	57	F	Precentral gyrus	L	–	DA	PR	none	GBM	GTR	TMZ, BEV RT 60 Gy	9.8	AWD
3	58	F	Inferior frontal gyrus	L	–	DA	GTR	none	GBM	GTR	TMZ, BEV RT 60 Gy	5.8	Dead
4	54	F	Insula	L	–	DA	PR	none	GBM	GTR	TMZ, RT 60 Gy	10.0	PF
5	67	F	Insula	L	–	DA	GTR	none	GSM	PR	TMZ, GK	28.7	Dead

*MRI* magnetic resonance imaging, *EOR* extent of resection, *DA* diffuse astrocytoma, *GTR* gross total resection, *PR* partial resection, *STR* subtotal resection, *TMZ* temozolomide, *RT* radiation therapy, *BEV* bevacizumab, *GK* gamma knife, *PFS* progression free survival, *AWD* alive with disease, *PF* progression free

Recurrent tumors developed in or near the surgical defects in all patients, and all tumors showed contrast enhancement on MRI. The second surgery was performed in all patients, at a mean of 12.4 months after the initial surgery (progression-free survival; PFS). The median overall survival (OS) of five patients was 28.7 months (95% CI, 19.0 to 38.4). A histopathological diagnosis of GBM was made in four patients and one of gliosarcoma in one patient (Table 1). As treatment at recurrence, all patients underwent adjuvant temozolomide and concurrent local radiotherapy or gamma knife radiosurgery. Two patients underwent a third surgery for recurrent tumors. Bevacizumab was additionally prescribed to three patients after the second or third surgery.

Table 2 shows the molecular characteristics of the initial and recurrent tumors. The status of *IDH1*, *IDH2*, *H3F3A*, *HIST1H3B*, and *BRAF* was “wild-type” in all patients. *TERT* promotor mutations (C250T or C228T) were detected in four patients. No tumors harbored 1p/19q codeletion. *EGFR* amplification, or chromosome 7 gain (+ 7) was detected in one patient at only recurrence. *CDKN2A/B* homozygous deletion was detected in two patient and *PTEN* losses were detected in one patients. According to these molecular details, four patients showed the high-grade molecular features of GBM as defined by the cIMPACT-NOW update 3 criteria^9^.

**Table 2:** Molecular alterations observed in five patients with initial and recurrent localized *IDH* wild-type histologically diffuse astrocytomas.

Patient No	Operation	*IDH1* mutation	*IDH2* mutation	Histone *H3F3A* mutation	*TERT* promoter mutation	BRAF V600E mutation	1p/19q co-deletion	*EGFR* amplification	*CDKN2A/B* homozygous deletion	*PTEN* loss	*TP53* loss	*CDK4* amplification	*MDM2* amplification	+ 7/− 10
1	1st	−	−	−	C250T	−	−	−	−	−	Hemi	−	−	−
	2nd	−	−	−	C250T	−	19q	−	−	−	Hemi	−	−	−
2	1st	−	−	−	C250T	−	−	−	−	−	Hemi	+	+	−
	2nd	−	−	−	C250T	−	−	+	−	−	−	+	+	+ 7
3	1st	−	−	−	C228T	−	−	−	+	−	−	−	−	−
	2nd	−	−	−	C228T	−	19q	−	+	−	Hemi	−	−	−
4	1st	−	−	−	−	−	19q	−	−	−	Hemi	−	−	−
	2nd	−	−	−	−	−	−	−	+	−	Hemi	−	−	−
5	1st	−	−	−	C228T	−	19q	−	−	−	Hemi	−	−	−
	3rd	−	−	−	C228T	−	19q	−	Hemi	+	−	−	−	−

*IDH* isocitrate dehydrogenase, *TERT* telomerase reverse transcriptase, *BRAF* v-raf murine sarcoma viral oncogene homolog B1, *EGFR* epidermal growth factor receptor, *CDKN2A/B* cyclin-dependent kinase inhibitor 2A/B, *PTEN* phosphatase and tensin homolog deleted on chromosome 10, *TP53* TUMOR PROTEIN p53, *CDK4* cyclin-dependent kinase 4, *MDM2* mouse double minute 2, + 7; whole chromosome 7 gain, − 10; whole chromosome 10 loss, *hemi*; hemizygous deletion.

### Case presentation (Case 3)

**Figure 3:**
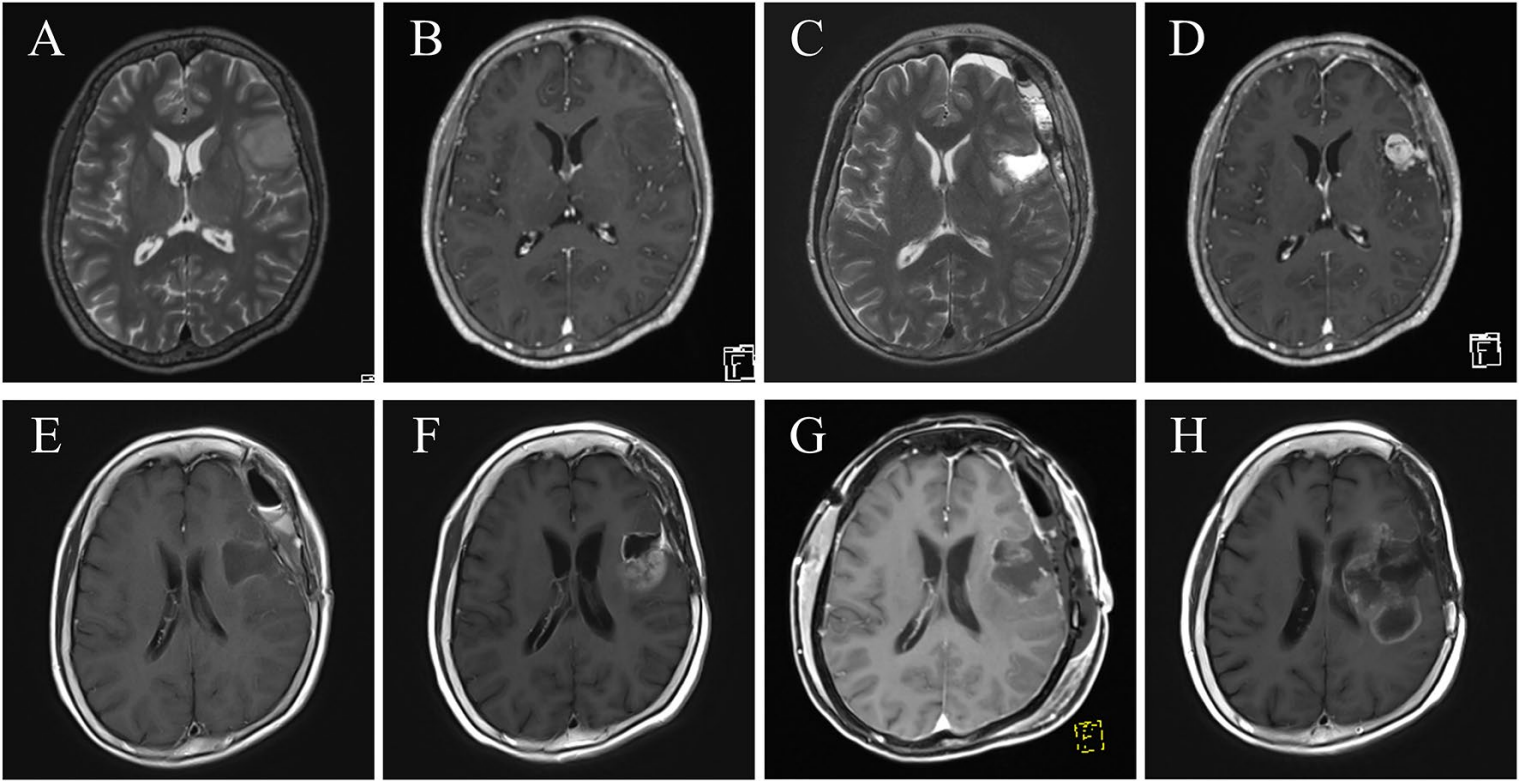
(**A**) Preoperative axial T2-weighted and (**B**) axial T1-weighted MRI with gadolinium enhancement, showing a high-intensity area with no enhancement in the left inferior frontal gyrus forming a localized tumor mass. (**C**) Postoperative axial T2-weighted MRI showing no tumors due to gross-total resection (first surgery). (**D**) Axial T1-weighted MRI with gadolinium enhancement performed about 6 months after the first surgery, showing nodular enhancement in the left frontal surgical cavity. (**E**) Postoperative axial T1-weighted MRI with gadolinium enhancement, showing no tumors due to gross-total resection (second surgery). (**F**) Axial T1-weighted MRI with gadolinium enhancement performed about 4 months after the second surgery, showing enhancing mass lesion in the surgical cavity again. (**G**) Postoperative axial T1-weighted MRI with gadolinium enhancement, showing no tumors owing to gross-total resection (third surgery). (**H**) Axial T1-weighted MRI with gadolinium enhancement at last follow-up, showing recurrent tumor. Tumor progression could not be controlled.

**Figure 4:**
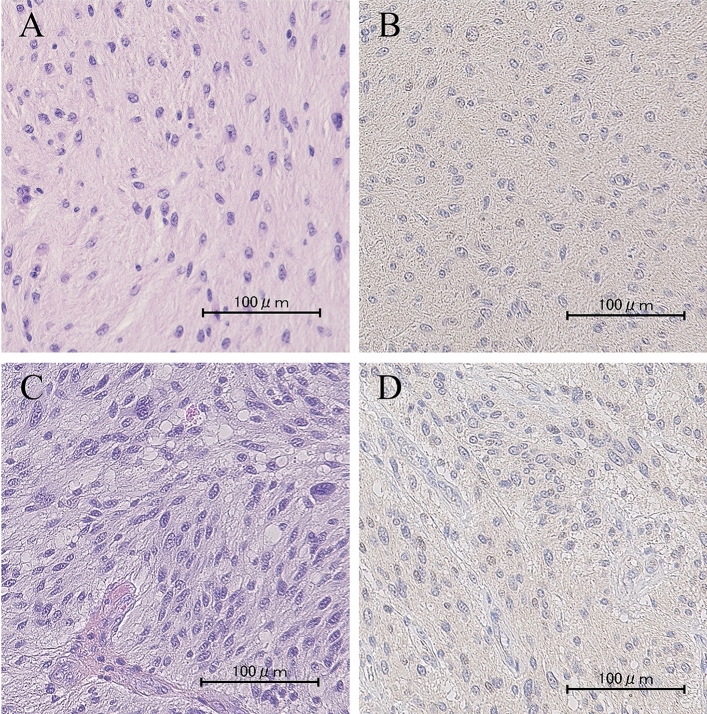
Histologic and immunohistochemical findings. (**A**) HE staining of initial surgery specimen showing diffuse infiltration of atypical astrocytes without necrosis or micro vascular proliferation. (**B**) IHC staining of IDH1 R132H of initial surgery specimen showing negative results. (**C**) HE staining of second surgery specimen showing proliferation of atypical glial cells with mitoses and microvascular proliferation. (**D**) IHC staining of IDH1 R132H of second surgery specimen showing negative results. Scale bars: 100 μm.

A 58-year-old woman presented with paresthesia of the right side of her body and visited a nearby hospital. She underwent brain MRI that revealed a T2-weighted high-intensity lesion, mainly in the left frontal lobe (Fig. 3A). The patient was referred to our hospital for further investigation and treatment. On her first visit to the outpatient clinic, she had no abnormal neurological findings, including palsy or aphasia. Brain MRI detected a low-intensity lesion on T1-weighted images with no gadolinium enhancement and a high-intensity lesion with relatively well-marked boundaries on T2-weighted images in the left inferior frontal gyrus, with a maximum diameter of approximately 30 mm (Fig. 3A,B). The patient underwent awake craniotomy assisted by both cortical and subcortical functional mapping. Functional mapping was performed with a double task combining picture-naming and right-arm movements. GTR was achieved (Fig. 3C). Postoperatively, the patient showed no remarkable neurological deterioration in language abilities and movement of the upper and lower extremities. The histopathological diagnosis was diffuse astrocytoma (Fig. 4A). Immunohistochemistry staining for IDH1 R132H was negative (Fig. 4B). Molecular analysis revealed the *TERT* promoter mutation C228T and *CDKN2A/B* homozygous deletion. No postoperative adjuvant therapy was administered.

Approximately 6 months later, follow-up contrast MRI showed a well-enhancing mass lesion in the left frontal surgical cavity (Fig. 3D). The patient underwent awake surgery for the recurrent tumor, and GTR was achieved (Fig. 3E). Histological examination of the tumor yielded a diagnosis of GBM, and Ki-67 expression was observed in 5–7% of the tumor cells (Fig. 4C). At the recurrent stage, immunohistochemical staining of IDH1 R132H was also negative (Fig. 4D).

Following the second surgery, the patient was treated with 60 Gy in 30 fractions of radiotherapy in combination with concurrent oral TMZ chemotherapy (standard Stupp protocol). Furthermore, the patient received a maintenance regimen of TMZ 200 mg/m^2^ on days 1–5 every 4 weeks. Four months after the second surgery, follow-up MRI revealed a recurrent lesion on the posterior side of the surgical defect, and bevacizumab was additionally prescribed (Fig. 3F).

However, the recurrent lesion showed progression, and a third craniotomy for tumor removal was performed 9.5 months after the second surgery; GTR was achieved (Fig. 3G). The histopathological diagnosis changed from diffuse astrocytoma to GBM. Stereotactic radiotherapy (40 Gy) was performed postoperatively, and chemotherapy, including temozolomide and bevacizumab, was continued. Despite multidisciplinary treatment, tumor progression was not controlled (Fig. 3H). The patient decided to leave the hospital to receive palliative care at home, and she died approximately 23 months after being diagnosed with diffuse astrocytoma.

## Discussion

In the current study, we present a single-institution retrospective series of five patients who were diagnosed with localized *IDH*wt histologically diffuse astrocytomas. We provide their clinical information, radiographic characteristics, genetic alterations, and treatments such as surgery and adjuvant therapy. The radiographical and pathological features of the initial tumors in our case series were almost identical to those of diffuse astrocytomas, *IDH*mut (CNS WHO grade 2). All tumors relapsed at a mean of 12.4 months after initial surgery, resulting in a dismal prognosis despite multidisciplinary treatment.

Among the five cases presented here, there were four patients with molecular features of GBM, which represents a new entity proposed by the cIMPACT-NOW committee to classify a subset of *IDH*wt diffuse or anaplastic astrocytomas into a “GBM, *IDH*wt, CNS WHO grade 4” category^12,18^. These four cases harbored *TERT* promoter mutations. *TERT* promoter mutations are known to be molecular markers of an aggressive phenotype, with which even LGGs behave like GBMs, and the pathological diagnosis of such cases is GBM-*IDH*wt, according to the 2021 WHO classification of tumors of the CNS^10^. Although these four tumors exhibited radiological and histopathological features similar to *IDH*mut, CNS WHO grade 2 tumors, they presented with an aggressive clinical course leading to poor survival, more similar to patients with both histological and molecular GBM, *IDH*wt, CNS WHO grade 4. Moreover, the exact prognosis of *IDH*wt LrGGs that do not meet the definition of molecular GBM is unknown. One retrospective study reported that the OS associated with *IDH*wt LrGGs not meeting the definition of molecular GBM is significantly longer than that associated with molecular GBMs^12^, while another study reported that there was no significant difference between the prognoses for these two groups^11^. Our patient (Case 4) did not meet the definition of molecular GBM and relapsed 10 months after partial resection during the initial surgery. The second pathological diagnosis was one of GBM. The clinical course of this patient did not seem to significantly differ from that of other cases of molecular GBM (Cases 1–3,5). The initial tumor of Case 4 may have been a lower-grade astrocytoma, not a GBM, at initial diagnosis, and it may have subsequently harbored *CDKN2A/B* homozygous deletions at recurrence 10 months after the first surgery.

Based on clinical data, radiographic imaging, and histopathology only, tumors with molecular features of GBM are difficult to distinguish from *IDH*mut LGGs. Although *IDH*wt histologically diffuse astrocytomas are uncommon, some studies have revealed that they are especially rare in women, and that they are associated with older age at initial diagnosis, fronto-temporo-insular location, and highly invasive behavior^3,5,7^ with frequent infiltration of the adjacent cortex and deep white matter^19^. Therefore, *IDH*wt histologically diffuse astrocytomas possess features of highly infiltrative patterns, such as gliomatosis cerebri growth patterns with widespread involvement^2^.

Data on tumor growth patterns in patients with *IDH*wt grade II or III LrGGs from studies published in the literature between 2018 and 2021 were extracted and compiled as shown in Table 3^11–14,17,20–23^. In the cIMPACT-NOW update 3 report^12^, Tesileanu et al. reported that a gliomatosis cerebri growth pattern of LrGGs was present in 35.8% of *IDH*wt astrocytomas, meeting the definition of molecular GBM. Furthermore, this pattern had a high frequency of 52.4% in *IDH*wt astrocytomas, meeting the definition of molecular GBM with only *TERT* promoter mutations. In their study of 47 patients with *IDH*wt diffuse astrocytomas, Berzero et al. found that infiltrative gliomatosis cerebri growth patterns were detected in 72% of *IDH*wt diffuse astrocytomas. In particular, these patterns were found in 73% of *IDH*wt diffuse astrocytomas, meeting the definition of molecular GBM^11^. In our series, five cases (45.4%) presented with a localized tumor mass without a multifocal or gliomatosis-cerebri pattern. Conversely, six patients (54.5%) presented with tumors exhibiting gliomatosis-cerebri growth patterns. From the point of view of the tumor border line, tumors with sharp or well-defined borders account for 0–33% and tumors with indistinct or ill-defined borders account for 38–100% of all tumors. In a systematic review and meta-analysis, Lent et al. reported that *IDH*wt astrocytomas showed significantly fewer sharp borders than *IDH*mut astrocytomas (29% and 50%, respectively)^15^. Thus, localized *IDH*wt lower grade astrocytomas, such as those described here, are considered rare. Therefore, even in a patient presenting with radiographic imaging findings suggestive of a localized tumor mass, physicians should remain aware of the possibility of lesions representing *IDH*wt histologically diffuse astrocytomas.

**Table 3:** Literatures about *IDH* wild-type lower grade gliomas.

Citation	Year	Study type	Molcular stratification	n(*IDH*-wt grade II/III gliomas)	Age	Sex (%)	Grade II vs. III (%)	Location (%)	Growth pattern (%)	Enhancement (%)	EOR (%)	Adjuvant (%)	Total mutations (%)	OS (months)
Tesileanu et al.^12^	2020	R	*IDH1/2* status, + 7/ − 10, *pTERT* mutation, *EGFR* amplification	71	58 [19–78]	Female: 33.8 Male: 66.2	II:63 III:20 NOS:17	Frontal: 49.3 Parietal: 50.7 Temporal74.6 Occipital: 25.4 Insula: 58.2 Basal ganglia: 47.8	Gliomatosis cerebri: 35.8 Multifocal: 9.0	N/A	Resection: 16.9 Biopsy: 83.1	CT: 19.7 RT: 25.4 CRT: 42.3none: 12.7	*pTERT*: 94 + 7/− 10: 59 *EGFR*: 24	23.8
Wijnenga et al.^20^	2018	R	*IDH1/2* status, *pTERT* mutation, + 7/ − 10q, *EGFR* amplification	23 (includes OD and OA histology)	61 [52–65]	Female: 26.1 Male: 73.9	II: 100	Frontal: 4.3 Parietal: 4.3 Temporal: 39.1 Occipital: 4.3 Insula: 0 Basal ganglia: 17.4 Gliomatosis cerebri: 30.4	Gliomatosis cerebri: 30.4 Others: 69.6	N/A	0–89: 91.3 90–99: 4.3 100: 4.3	CT: 26.1 RT: 60.9 CRT: 8.7 none: 4.3	N/A	25.2
Ding et al.^13^	2019	R	*IDH1/2* status, *MGMT* methylation, *pTERT* mutation	28	39	Female: 21.4 Male: 78.6	N/A	Frontal: 7.1 Temporal: 7.1 Insula: 7.1 Brain stem: 28.6 Others: 50	Border well defined: 33 ill defined: 67	Yes: 33 no: 67	N/A	N/A	N/A	N/A
Villanueva-Meyer et al.^17^	2018	R	*IDH1/2* status	22	58	N/A	II: 100	Lobar: 73 Central: 27 Brainstem: 27	Multicentric: 5 Multifocal: 45	Yes: 27 no: 73	N/A	N/A	N/A	N/A
Park et al.^14^	2018	R	*IDH1/2* status	73	46.73 ± 15.79	Female: 54.8 Male: 45.2	II: 21.9 III: 78.1	Frontal: 39.7 Parietal: 15.1 Temporal: 17.8 Occipital: 1.4 Insula: 1.4	Multifocal/Multicentric: 32.9 Infiltrative: 52.1	Yes: 67.1 no: 32.9	N/A	N/A	N/A	N/A
Berzero et al.^11^	2021	R	*IDH1/2* status, + 7/ − 10, *pTERT* mutation, *EGFR* amplification	47	55	Female: 23 Male: 77	II: 100	Fronto-temporo-insular: 60 Fronto-callosal or parieto-callosal: 9 Thalamo-mesennphalic: 9	Infiltrative: 72 Nodular: 28	N/A	GTR: 18 PR: 20 Biopsy: 61	CT: 34 RT: 3 CRT: 32	*pTERT*: 51 *EGFR*: 9 7 + /10−: 17	59.1
Delfanti et al.^21^	2017	R	*IDH1/2* status	13	N/A	N/A	N/A	Frontal: 15.3 Non-frontal: 84.7	Border well-dfined: 15.3 ill-defined: 38.4	< 25%: 69.2 25–75%: 30.8	N/A	N/A	N/A	N/A
Yamauchi et al.^22^	2018	R	*IDH1/2* status	30	53.5 [5–76]	Female: 40.0 Male: 60.0	II: 33 III: 67	Frontal: 52.8 Temporal: 11.1 Parietal: 11.1 Occipital: 2.8 Multiple: 22.2	Margin clear: 38.9 indistinct: 61.1	Yes: 47.2 no: 52.8	N/A	N/A	N/A	N/A
Hyare et al.^23^	2019	R	*IDH1/2* status	52	54 [21–76]	Female: 18 Male: 34		Frontal: 25.0 Temporal: 28.8 Parietal: 9.6 Occipital: 1.9 Insula: 7.7 Thalamus: 21.2	Gliomatosis cerebri: 21.2 Multifocal: 17.3	Avid: 17.3 Mild: 34.6 no: 46.2	N/A	N/A	N/A	N/A

*IDH* isocitrate dehydrogenase, *wt* wild-type, *EOR* extent of resection, *OS* overall survival, *R* retrospective study, *pTERT* telomerase reverse transcriptase promoter, *EGFR* epidermal growth factor receptor, *NOS* not otherwise specified, *N/A* not available, *CT* chemotherapy, *RT* radiotherapy, *CRT* chemoradiotherapy, *OD* oligodendroglioma, *OA* oligoastrocytoma, *GTR* gross total resection, *PR *partial resection.

Surgery of localized tumors tends to achieve greater extent of resection (EOR) than resection for infiltrative tumors, and it has been reported that a greater EOR is associated with better outcomes for *IDH*wt LrGGs^24,25^. Moreover, some studies have reported that *IDH*wt grade II gliomas have a better prognosis than *IDH*wt grade III gliomas^5,11,26^. While this suggests that localized *IDH*wt grade II gliomas also have a better prognosis, contrary to our expectations, our cases showed an extremely aggressive clinical course. In Cases 1 and 3, the tumor relapsed only 7.5 and 5.8 months after initial surgery, even after GTR had been achieved. All recurrent tumors in our cases developed in or near the surgical defects and retained their localized radiological features. In four cases (Cases 1–3, 5), recurrent tumors showed resistance to multidisciplinary treatment and resulted in progression. Only one case (Case 4) was undergoing a maintenance regimen of TMZ without progression. Our findings suggest that *IDH*wt histologically diffuse astrocytomas need to be followed up very cautiously, even after gross total removal, and that strong postoperative adjuvant therapy such as the Stupp protocol for GBM should be considered.

Taken together, there is a strong need for routine analysis for molecular features of GBM, even for seemingly low-grade components. Several studies have reported on non-invasive imaging modalities to predict *IDH* or *TERT* promoter mutation status to guide treatment strategies for gliomas from the preoperative stage of the initial clinical diagnosis.^27,28^ To predict the *IDH* status of gliomas from preoperative MRI, Choi et al.^28^ developed a model based on deep learning and radiomics using a fully automated hybrid approach. The authors demonstrated that their approach allows for the accurate prediction of *IDH* status of gliomas. Lu et al.^27^ presented a radiomics feature-based nomogram for predicting *TERT* promoter mutation status from preoperative MRI in patients with lower-grade glioma. The radiomics signature yielded good performance for predicting *TERT* promoter mutation status, with a high area under the curve (AUC) of 0.900 and 0.873 in the training and validation datasets, respectively. These modalities provide promising methods for preoperatively predicting molecular features of gliomas and have the potential to guide treatment strategies for patients with gliomas.

Although we provide novel information on localized *IDH*wt histologically diffuse astrocytomas, our results are somewhat limited, compared to those of prospective studies, as retrospective studies may be influenced by unrecognized biases. Moreover, the present study was based on a small number of tumor cases; therefore, a larger cohort study is needed to assess the clinical, radiographic, histopathological, and molecular characteristics of localized *IDH*wt histologically diffuse astrocytomas. Thus, further accumulation of evidence for *IDH*wt histologically diffuse astrocytomas will help improve the treatment of this disease and hopefully enable us to develop it into a novel therapy in the future.

Another limitation of this study is that the initial radiological diagnosis of diffuse astrocytoma could have been changed to high-grade glioma, including molecular features of GBMs, using advanced MRI techniques such as diffusion, perfusion, spectroscopy, tractography, and functional MRI^29^. Moreover, another limitation of our study was that we were unable to perform a second MRI approximately 6–12 weeks after the first diagnosis. This would have allowed for the detection of a high-grade glioma the radiological tumor growth rate, which is a strong indicator of tumor aggressiveness^30^. Repeat MRI could detect the tumor's features, even if the initial MRI presentation was in favor of low-grade gliomas. Thus, "multimodality" imaging should have been performed after the initial diagnosis to allow for a revision of the initial diagnosis of low-grade gliomas to high-grade gliomas.

## Conclusions

Although *IDH*wt histologically diffuse astrocytomas possess highly infiltrative patterns, such as gliomatosis cerebri growth patterns with widespread involvement, we observed clinical cases of localized *IDH*wt histologically diffuse astrocytomas, which resulted in malignant recurrence and a poor clinical prognosis similar to that of GBMs. Even in patients with histologically diffuse astrocytomas and those who present with radiographic imaging findings suggestive of a localized tumor mass, physicians should consider the possibility of *IDH*wt histologically diffuse astrocytomas.

## Materials and methods

### Patient data

We have totally experienced eight cases of *IDH*wt diffuse astrocytoma and thirteen cases of *IDH*mut diffuse astrocytoma between July 2017 and January 2020 at Nagoya University Hospital (Nagoya, Japan). Of these, the data of five patients who were diagnosed and treated for localized *IDH*wt diffuse astrocytoma were retrospectively retrieved from patient records. A localized tumor mass was defined as one with a well-defined tumor border, where the border between the tumor and normal brain can be delineated on T2-weighted MRI. Moreover, “localized tumors” do not present with gliomatosis cerebri growth patterns with widespread involvement or multifocal disease. Surgery was proposed with the aim of maximal tumor resection and preservation of neurological functions. In all five patients, we performed awake brain mapping with direct electrical stimulation using an asleep-awake-asleep protocol, as previously described^31–36^. Patient data on clinical information and outcomes, including age, sex, histopathological findings, prescribed treatment, EOR, radiographic findings before and after treatment, molecular features of the tumors, PFS and OS were collected and analyzed. Volumetric EOR was categorized as follows: GTR, EOR = 100%; subtotal resection (STR), EOR ≥ 90% to < 100%; and PR, EOR < 90%. Histopathological diagnoses had been established independently, by histological confirmation according to the 2016 WHO criteria^2^, by two expert neuropathologists (Y.S, M. N).

### Direct DNA sequencing and multiplex ligation-dependent probe amplification

Sanger sequencing was performed to detect mutations in *IDH1*, *IDH2*, *H3F3A*, *HIST1H3B*, *BRAF*, and *TERT* promoter genes, as previously described^37^. Multiplex ligation-dependent probe amplification (MLPA) was performed to detect copy number variations of chromosome 1p/19q codeletion, *EGFR* amplification, *CDKN2A/B* homozygous deletion, *PTEN*, *p53*, chromosome 7 gain, and chromosome 10 loss (7 + /10−).

### Statistical analyses

All statistical analyses were conducted using SPSS (version 27.0; IBM Corporation, Armonk, NY, USA) for Windows (Microsoft Corporation, Redmond, WA, USA). PFS was determined, from the day of the first surgery until the occurrence of true tumor progression before the second surgery. OS was calculated from the date of the first visit to the date of death or last follow-up.

### Ethics approval and consent to participate

The Ethics Committee of Nagoya University Hospital approved the data evaluation (Approval Number: 2021-0410). All procedures performed in studies involving human participants in accordance with all provisions of the Declaration of Helsinki. Patient informed consent was waived due to the retrospective nature of the study approved by the Ethics Committee of Nagoya University Hospital.

## Data Availability

The data in the current study are available from the corresponding author upon reasonable request.

## References

[1] Giese A, Bjerkvig R, Berens ME, Westphal M (2003). Cost of migration: Invasion of malignant gliomas and implications for treatment. J. Clin. Oncol..

[2] Louis DN (2016). The 2016 world health organization classification of tumors of the central nervous system: A summary. Acta Neuropathol..

[3] Metellus P (2010). Absence of IDH mutation identifies a novel radiologic and molecular subtype of WHO grade II gliomas with dismal prognosis. Acta Neuropathol..

[4] Wijnenga MMJ (2017). Molecular and clinical heterogeneity of adult diffuse low-grade IDH wild-type gliomas: Assessment of TERT promoter mutation and chromosome 7 and 10 copy number status allows superior prognostic stratification. Acta Neuropathol..

[5] Aibaidula A (2017). Adult IDH wild-type lower-grade gliomas should be further stratified. Neuro Oncol..

[6] Tabouret E (2016). Prognostic impact of the 2016 WHO classification of diffuse gliomas in the French POLA cohort. Acta Neuropathol..

[7] Cancer Genome Atlas Research, N (2015). Comprehensive, integrative genomic analysis of diffuse lower-grade gliomas. N. Engl. J. Med..

[8] Suzuki H (2015). Mutational landscape and clonal architecture in grade II and III gliomas. Nat. Genet..

[9] Brat DJ (2018). cIMPACT-NOW update 3: Recommended diagnostic criteria for "diffuse astrocytic glioma, IDH-wildtype, with molecular features of glioblastoma, WHO grade IV". Acta Neuropathol..

[10] Louis DN (2021). The 2021 WHO classification of tumors of the central nervous system: A summary. Neuro Oncol.

[11] Berzero G (2021). IDH-wildtype lower-grade diffuse gliomas: The importance of histological grade and molecular assessment for prognostic stratification. Neuro Oncol..

[12] Tesileanu CMS (2020). Survival of diffuse astrocytic glioma, IDH1/2 wildtype, with molecular features of glioblastoma, WHO grade IV: A confirmation of the cIMPACT-NOW criteria. Neuro Oncol..

[13] Ding H (2019). Prediction of IDH status through MRI features and enlightened reflection on the delineation of target volume in low-grade gliomas. Technol. Cancer Res. Treat..

[14] Park YW (2018). Prediction of IDH1-mutation and 1p/19q-codeletion status using preoperative MR imaging phenotypes in lower grade gliomas. AJNR Am. J. Neuroradiol..

[15] van Lent DI, van Baarsen KM, Snijders TJ, Robe P (2020). Radiological differences between subtypes of WHO 2016 grade II–III gliomas: A systematic review and meta-analysis. Neurooncol Adv..

[16] Lee D, Riestenberg RA, Haskell-Mendoza A, Bloch O (2021). Diffuse astrocytic glioma, IDH-Wildtype, with molecular features of glioblastoma, WHO grade IV: A single-institution case series and review. J. Neurooncol..

[17] Villanueva-Meyer JE (2018). MRI features and IDH mutational status of grade II diffuse gliomas: Impact on diagnosis and prognosis. AJR Am. J. Roentgenol..

[18] Weller M (2015). Molecular classification of diffuse cerebral WHO grade II/III gliomas using genome- and transcriptome-wide profiling improves stratification of prognostically distinct patient groups. Acta Neuropathol..

[19] Izquierdo C (2019). Radiological characteristics and natural history of adult IDH-wildtype astrocytomas with TERT promoter mutations. Neurosurgery.

[20] Wijnenga MMJ (2018). The impact of surgery in molecularly defined low-grade glioma: An integrated clinical, radiological, and molecular analysis. Neuro Oncol..

[21] Delfanti RL (2017). Imaging correlates for the 2016 update on WHO classification of grade II/III gliomas: Implications for IDH, 1p/19q and ATRX status. J. Neurooncol..

[22] Yamauchi T (2018). Radiological characteristics based on isocitrate dehydrogenase mutations and 1p/19q codeletion in grade II and III gliomas. Brain Tumor Pathol..

[23] Hyare H (2019). Modelling MR and clinical features in grade II/III astrocytomas to predict IDH mutation status. Eur. J. Radiol..

[24] Patel T (2018). The role of extent of resection in IDH1 wild-type or mutant low-grade gliomas. Neurosurgery.

[25] Wang P, Luo C, Hong PJ, Rui WT, Wu S (2021). The role of surgery in IDH-wild-type lower-grade gliomas: Threshold at a high extent of resection should be pursued. Neurosurgery.

[26] Aoki K (2018). Prognostic relevance of genetic alterations in diffuse lower-grade gliomas. Neuro Oncol..

[27] Lu J, Li X, Li H (2022). A radiomics feature-based nomogram to predict telomerase reverse transcriptase promoter mutation status and the prognosis of lower-grade gliomas. Clin. Radiol..

[28] Choi YS (2021). Fully automated hybrid approach to predict the IDH mutation status of gliomas via deep learning and radiomics. Neuro Oncol..

[29] Villanueva-Meyer JE, Mabray MC, Cha S (2017). Current clinical brain tumor imaging. Neurosurgery.

[30] Pallud J (2006). Prognostic value of initial magnetic resonance imaging growth rates for World Health Organization grade II gliomas. Ann. Neurol..

[31] Duffau H, Peggy Gatignol ST, Mandonnet E, Capelle L, Taillandier L (2008). Intraoperative subcortical stimulation mapping of language pathways in a consecutive series of 115 patients with Grade II glioma in the left dominant hemisphere. J. Neurosurg..

[32] Motomura K (2017). Surgical benefits of combined awake craniotomy and intraoperative magnetic resonance imaging for gliomas associated with eloquent areas. J. Neurosurg..

[33] Motomura K (2018). Supratotal resection of diffuse frontal lower grade gliomas with awake brain mapping, preserving motor, language, and neurocognitive functions. World Neurosurg..

[34] Motomura K (2020). Navigated repetitive transcranial magnetic stimulation as preoperative assessment in patients with brain tumors. Sci. Rep..

[35] Motomura K (2021). Impact of the extent of resection on the survival of patients with grade II and III gliomas using awake brain mapping. J. Neurooncol..

[36] Motomura K (2019). Neurocognitive and functional outcomes in patients with diffuse frontal lower-grade gliomas undergoing intraoperative awake brain mapping. J. Neurosurg..

[37] Motomura K (2011). Benefits of interferon-beta and temozolomide combination therapy for newly diagnosed primary glioblastoma with the unmethylated MGMT promoter: A multicenter study. Cancer.

